# Wheat Flour Substitution with Sacha Inchi Tea Leaf Powder: Effects on Physicochemical Properties, Starch Digestibility, and Sensory Attributes of Cookies

**DOI:** 10.3390/foods15162801

**Published:** 2026-08-11

**Authors:** Praew Chantarasinlapin, Kunyakorn Kamonkunakasem, Titima Wongdee, Arkapol Phunkhow, Ampornphat Sudtiastasilp, Charoonsri Chusak, Sirichai Adisakwattana

**Affiliations:** Center of Excellence in Phytochemical and Functional Food for Clinical Nutrition, Department of Nutrition and Dietetics, Faculty of Allied Health Sciences, Chulalongkorn University, Bangkok 10330, Thailand; praew.c@chula.ac.th (P.C.); ka.kunyakorn@gmail.com (K.K.); titimawongdee1@gmail.com (T.W.); mrppodd2288@gmail.com (A.P.); manyampornphat@gmail.com (A.S.); charoonsri.c@chula.ac.th (C.C.)

**Keywords:** *Plukenetia volubilis*, bakery products, functional ingredient, antioxidant capacity, predicted glycemic index, consumer acceptance

## Abstract

Sacha inchi *(Plukenetia volubilis* L.) leaves are an underutilized by-product of an expanding oilseed crop, yet their use as a food ingredient remains limited. This study evaluated sacha inchi tea leaf powder (STLP) as a partial replacement for wheat flour in cookies at 0%, 2.5%, 5%, and 10% (*w*/*w*), and examined the physicochemical properties, antioxidant capacity, in vitro starch digestibility, and sensory acceptability of the cookies. Substitution with STLP significantly increased the protein, dietary fiber, and ash contents of the cookies (*p* ≤ 0.05). Total phenolic content and ferric-reducing antioxidant power also increased with the substitution level, particularly at 5% and 10% STLP (*p* ≤ 0.05). In the simulated digestion model, STLP-substituted cookies showed lower glucose release, glucose area under the curve, hydrolysis index, and predicted glycemic index than the control (*p* ≤ 0.05), indicating reduced starch hydrolysis in vitro. Physical properties were largely maintained at low to moderate substitution levels, whereas 10% STLP increased spread ratio and hardness, and reduced baking loss. Sensory evaluation showed that STLP substitution lowered appearance, color, smell, flavor, and overall acceptability scores compared with the control, while texture scores were not significantly affected. These findings suggest that STLP may be used as a potential ingredient to improve protein, fiber and ash contents, enhance antioxidant properties of cookies, and reduce in vitro starch hydrolysis. Although further formulation optimization is needed to balance these effects with sensory acceptability, STLP is a promising functional ingredient for bakery products and a means of valorizing an underutilized agricultural material.

## 1. Introduction

Sacha inchi (*Plukenetia volubilis* L.) is categorized as an oilseed species originating in the Amazon, increasingly regarded as a promising yet underexploited crop [1]. Its seeds are the main commercial product, pressed for oil or eaten as nuts because of their high content of unsaturated fatty acids and protein [1,2]. Its cultivation has expanded from South America into parts of Asia, including Thailand. The planted area and farm-gate price of sacha inchi are now recorded in Thailand’s national agricultural statistics, indicating its increasing agricultural and economic relevance [3].

Commercial and scientific attention has focused on the seeds and oil, while the leaves have remained comparatively neglected [2,4]. The leaves are used mainly fresh or as a dried herbal tea sold in local markets, suggesting a possible route for value addition, although their broader application as a food ingredient remains limited. Sacha inchi leaves contain phenolic compounds and exhibit antioxidant activity [4]. A previous study reported that oven-dried sacha inchi leaves contained total phenolic content ranging from 12.82 to 13.10 mg gallic acid equivalent (GAE)/g dry weight and exhibited ferric reducing antioxidant power (FRAP) of 45.70–48.26 µmol Trolox equivalent/g dry weight [4]. A previous in vitro study has also reported inhibition of carbohydrate-digesting enzymes, including pancreatic α-amylase and α-glucosidase, by sacha inchi leaf extracts [4]. Together, these observations suggest that the leaves could serve as a plant-derived ingredient for enhancing antioxidant-related properties and modulating starch digestion in food matrices. However, such effects need to be evaluated within actual food formulations, as ingredient functionality can be modified by processing conditions and matrix interactions. Broadening the use of sacha inchi leaves could improve the economic return from the crop and support the valorization of an underutilized agricultural material.

Cookies are a widely consumed bakery product because of their convenience and palatability, and extended shelf life. However, cookies are typically prepared with refined wheat flour, fat, and sugar, and often contain limited amounts of dietary fiber and micronutrients. Accordingly, there is growing interest in reformulating cookies with plant-derived materials, such as leaf, seed, and fruit by-products, which can improve their nutritional profile without compromising acceptable product quality [5,6,7,8,9,10,11]. Substituting a portion of wheat flour with fiber- and phenolic-rich plant materials is a practical strategy to modify the composition profile of cookies while creating new applications for underexploited raw materials. Plant leaf powders are increasingly used to enrich cookies, and evaluating their effects on physical quality, antioxidant properties, starch digestibility, and sensory acceptability is essential for determining feasible substitution levels [6,9]. Beyond nutritional enrichment, dietary fiber and phenolic compounds may contribute to slower starch hydrolysis by limiting enzyme accessibility, altering matrix structure, or interacting with digestive enzymes, although these mechanisms are formulation-dependent [12,13].

To our knowledge, the incorporation of sacha inchi tea leaf powder (STLP) into cookies has not been reported. Therefore, the study aimed to examine how replacing wheat flour with STLP at different levels (0–10% *w*/*w*) affected the physicochemical properties, antioxidant capacity, in vitro starch digestibility, and sensory acceptability of cookies. The findings are expected to provide preliminary formulation evidence for using STLP as an underutilized plant ingredient in bakery products, while identifying the balance between compositional improvement and sensory acceptability.

## 2. Materials and Methods

### 2.1. Chemicals and Reagents

Sacha inchi tea leaves (*Plukenetia volubilis* L.) were purchased from the local vendor in Chiang Rai, Thailand. The following reagents were supplied by Sigma-Aldrich Chemical (St. Louis, MO, USA): Folin–Ciocalteu reagent, 2,4,6-tripyridyl-s-triazine (TPTZ; 93285), porcine pancreatic α-amylase (Type VI-B; ≥5 units/mg solid; A3176), porcine pepsin (≥250 units/mg solid; P7000), porcine pancreatin (4 × USP specifications; P1750), and porcine bile extract (B8631). Amyloglucosidase (3260 units/mL; E-AMGDF) was obtained from Megazyme (Bray, Ireland). Enzyme activities were based on the values declared by the manufacturers, and the amounts added to the digestion mixtures were calculated accordingly. The enzyme activities were not independently verified before the experiments. Glucose LiquiColor^®^ was supplied by HUMAN GmbH (Wiesbaden, Germany).

### 2.2. Preparation of Sacha Inchi Tea Leaf Powder (STLP)

Commercially processed, loose-leaf dried sacha inchi leaves prepared from mature foliage were obtained from a local supplier in Chiang Rai, Thailand. The material was supplied in dried form and was not prepared by aqueous extraction. According to the supplier’s product information, it consisted of sacha inchi leaves only, without shells or other plant parts. Following a previously established protocol [14], the dried leaves were milled using a tilted-head stand mixer (DXM-500, DXFill Machine, Samut Prakan, Thailand) and sieved through a 150-µm screen. The prepared sample was then kept in airtight foil pouches and maintained at −20 °C prior to testing.

### 2.3. Cookie Preparation

The cookie formulation was developed specifically for this study through preliminary formulation trials. All-purpose flour was substituted with STLP at four levels: 0% (control), 2.5%, 5%, and 10% (*w*/*w*). The substitution levels were consistent with substitution ranges used in previous studies on leaf powder-supplemented cookies [10,15]. In the preliminary trials, substitution above 10% produced a dough that was too dense and stiff to be pressed using a cookie cutter. Therefore, 10% STLP substitution was adopted as the upper limit.

For the control formulation, the cookie dough contained white wheat flour (80 g), rice-bran oil shortening (40 g), caster sugar (30 g), egg (15 g), matcha flavor (4 g), baking powder (1 g) and salt (0.5 g). For STLP-containing formulations, wheat flour was replaced with STLP on a weight basis according to the assigned substitution level, while the amounts of the other ingredients were kept constant. All ingredients were thoroughly blended using a stand mixer (5K45SS Heavy Duty, KitchenAid, Greenville, OH, USA) to obtain a uniform dough. Following refrigeration for 15 min, the cookie dough was shaped into individual pieces approximately 4.5 cm in diameter, 0.5 cm thick, and 7 g in weight using a cookie cutter. All cookie formulations were baked in the same oven (S.p.A., Nardi Elettrodomestici, Bordj Bou Arréridj, Algeria) at 170 °C for 12 min under identical conditions. The baked cookies were cooled at an ambient laboratory temperature of 24–26 °C for at least 30 min before subsequent analyses. Three independent dough preparation and baking batches were produced for each formulation, with each batch considered an experimental replicate.

### 2.4. Proximate Analysis

A representative sample of STLP was submitted to an external food-analysis laboratory for proximate characterization. Ash and moisture contents were determined according to the Association of Official Analytical Chemists (AOAC) methods 925.51A [16] and 2008.60 [17] , respectively. Protein content was determined using a method based on AOAC 981.10 [17], applying a standard multiplier of 6.25 to convert total nitrogen to protein. Total dietary fiber was analyzed using a method based on AOAC 985.29 [17], while fat content was determined using a method based on AOAC 922.06 [17]. Carbohydrate and energy values excluding dietary fiber were calculated according to the methods of analysis for nutrition labeling [18]. The STLP contained 8.06 g/100 g moisture, 17.8 g/100 g protein, 33.4 g/100 g total dietary fiber, 11.7 g/100 g ash, less than 0.01 g/100 g fat, 29.0 g/100 g carbohydrate excluding dietary fiber, and 254 kcal/100 g energy excluding dietary fiber.

Using a standard protocol from AOAC [17], the samples were analyzed for moisture, protein, total fat, total dietary fiber, and ash fractions. Total carbohydrate (by difference) and energy content were then calculated based on the methods of analysis for nutrition labeling [18].

### 2.5. Spread Ratio and Baking Loss

Spread ratio was measured following the previous method [19] with slight modifications. Six cookies were arranged edge to edge, and their total length was measured with a ruler. The cookies were rotated 90° and re-measured, and the diameter was taken as the mean of the two readings divided by six. For thickness, six cookies were stacked, and the stack height was measured using a vernier caliper. The stack was then reassembled in a random order and re-measured. The thickness was taken from the mean of the two readings divided by six. Spread ratio was expressed as mean diameter divided by mean thickness. Baking loss was evaluated as the mass reduction during thermal processing, determined by comparing pre- and post-baking cookie weights, reported as a percentage of the initial dough weight. All measurements were performed in three independent batches.

### 2.6. Hardness Measurement

Hardness was measured based on the previous method [20] with slight adjustment. A texture analyzer (TA.XTplusC, Stable Micro Systems, Surrey, UK) fitted with a 50 kg load cell and a 6 mm cylindrical probe (P6) was used. The trigger force was set to 5 g. After calibration, measurements were carried out at a pre-test speed of 2 mm/s, a test speed of 0.5 mm/s, a post-test speed of 10 mm/s, and a penetration depth of 7 mm. Hardness was quantified as the maximum force recorded during probe penetration into the cookie. It was selected as the primary texture parameter, as instrumental hardness has been shown to correlate with the sensory perception of texture in brittle cookie-type foods [21]. This is consistent with recent studies on plant-powder-substituted cookies, which similarly used peak force during probe penetration as the primary texture indicator [22]. Twenty cookies were tested per formulation, with one measurement taken per cookie.

### 2.7. Color Measurement

The color of cookies was assessed with a colorimeter (Color-flex EZ, Hunter Lab, Reston, VA, USA), following a previous study [14] with modification. Prior to measurement, randomly selected cookies were homogenized using a food processor (5KFC3516C KitchenAid, Benton Harbor, MI, USA). Homogenized samples were used rather than the intact surface to minimize variability from position-dependent surface heterogeneity, such as uneven browning between the edge and center of the cookie. Measurements were taken in triplicate and reported as L* (lightness), a* (ranging from greenness, −a*, to redness, +a*), and b* (ranging from blueness, −b*, to yellowness, +b*). The color difference (∆*E*) between the control and STLP-substituted cookies was calculated using the following equation [7]:

(1) $$∆E=\sqrt{(L_{0}^{*}{-L^{*})}^{2}+(a_{0}^{*}{-a^{*})}^{2}+(b_{0}^{*}{-b^{*})}^{2}}$$

where *L*_0_*, *a*_0_*, *b*_0_* represent the values of the control and L*, a*, b* represent those of the STLP-substituted cookies.

### 2.8. Total Phenolic Content (TPC)

A simple aqueous extraction was used to enable a practical comparison of phenolic content across formulations. Cookie samples were ground to coarse powder and mixed with distilled water (1:10 *w*/*v*) for 2 h at 20 rpm on a shaker (Mini-Rocker Shaker MR-1, Biosan, Riga, Latvia), after which the mixture was filtered through Whatman No. 1 filter paper (125 mm). TPC was determined following the published procedure [23]. A 50 µL aliquot of the filtrate was combined with 50 µL of Folin–Ciocalteu reagent (10% *w*/*v*) and incubated in the dark at room temperature for 10 min. Subsequently, 50 µL of Na_2_CO_3_ (10% *w*/*v*) was added, and the mixture was again incubated in the dark at room temperature for another 10 min. Absorbance was then read at 760 nm on a microplate reader (PowerWave XS2, BioTek, Winooski, VT, USA). Levels of TPC were reported as mg GAE/g sample. The assay was performed in triplicate for each formulation.

### 2.9. Ferric-Reducing Antioxidant Power (FRAP)

The antioxidant capacity of the cookie samples was evaluated by the FRAP assay following [24]. Sodium acetate buffer (0.3 M, pH 3.6), TPTZ (10 mM in 40 mM HCl), and FeCl_3_ (20 mM) were combined at a 10:1:1 ratio to prepare fresh FRAP reagent. A 20 µL sample extract was then reacted with 180 µL of the FRAP reagent and held in the dark at room temperature for 30 min. The mixture was centrifuged at 10,000 rpm for 10 min at 4 °C (ROTINA 380R, Hettich, Tuttlingen, Germany). The absorbance of the supernatant was read at 595 nm on a microplate reader (PowerWave XS2, BioTek, Winooski, VT, USA). The results were reported as mM FeSO_4_ equivalent/g sample. Each cookie formulation was evaluated in three independent replicates.

### 2.10. Starch Digestion

In vitro simulated digestion of the cookie samples was carried out following the previous procedure [23] with slight modification. For the oral phase, ground cookie samples (500 mg) were combined with porcine α-amylase (250 U/mL) in 0.2 M carbonate buffer (pH 7) and incubated for 15–20 s at 37 °C in a water bath shaker at 100 rpm (NB-304, N-BIOTEK, Gyeonggi, Republic of Korea). For the gastric phase, pepsin solution (1 mg/mL in 0.02 M HCl) was added, then the pH was adjusted to 2. The mixture was incubated at 37 °C for 30 min before neutralization with 0.02 M NaOH and 0.2 M sodium acetate buffer (pH 6). For the small intestinal phase, a solution of pancreatin (2 mg/mL) and 10% bile extract was added, and the incubation continued at 37 °C in the shaker. Aliquots were collected at 0, 15, 20, 30, 45, 60, 90, 120, and 180 min, heated at 100 °C for 10 min to inactivate the enzymes, and centrifuged at 5000 rpm for 15 min at 4 °C (ROTINA 380R, Hettich, Tuttlingen, Germany). The supernatant was retained and kept at −20 °C until subsequent analysis.

Digesta glucose concentration was quantified by the GOPOD enzymatic colorimetric method using Glucose LiquiColor^®^ (HUMAN GmbH, Wiesbaden, Germany). The digesta sample was mixed with the working reagent in a 1:100 proportion and incubated at room temperature for 10 min. A microplate reader (PowerWave XS2, BioTek, Winooski, VT, USA) was used to measure absorbance at 500 nm. A glucose standard (100 mg/dL) was used to determine the glucose released at each digestion time point. The glucose area under the curve (AUC) was obtained by the trapezoidal rule. The hydrolysis index (HI) and predicted glycemic index (pGI) of the cookie samples were calculated using the following equations [14,25,26]:

(2) HI = (AUC_sample_/AUC_glucose_) × 100

(3) pGI = 39.71 + 0.549 HI

### 2.11. Sensory Evaluation

An affective test was conducted using a 9-point hedonic scale to evaluate consumer acceptance of the cookies. A total of 50 untrained panelists (9 males, 41 females, mean age 25.72 ± 6.73 years) were recruited by convenience sampling. Eligibility criteria were as follows: aged 18–59 years; having a normal sense of taste and smell, and not colorblind; free from serious illness such as heart disease, kidney disease, or cancer; no history of allergy to any ingredient used in the study cookies; not pregnant or breastfeeding; and voluntary participation with informed consent.

Sensory evaluation was performed in isolated testing booths to minimize interaction among panelists. Samples were labeled with three-digit random codes to minimize bias, and panelists rinsed their mouths with water between samples. The panelists evaluated the appearance, color, texture, smell, flavor, and overall acceptability of the cookies, where 1 represented “extremely dislike,” 5 represented “neither like nor dislike,” and 9 represented “extremely like.” The study protocol was approved by the Research Ethics Review Committee for Research Involving Human Research Participants, Group 1, Chulalongkorn University (COA No. 180/66, approved 29 August 2023). All participants provided informed consent before sensory evaluation.

### 2.12. Statistical Analyses

Results were reported as means ± standard error of the mean (SEM). Prior to statistical comparison, the normality of data distribution was assessed using the Shapiro–Wilk test alongside visual inspection of histograms and Q-Q plots. Homogeneity of variance was evaluated using Levene’s test. Group means were compared by one-way ANOVA with Tukey’s HSD post hoc test, with statistical significance accepted at *p* ≤ 0.05. All analyses were carried out in SPSS (Version 29, IBM, Chicago, IL, USA).

## 3. Results and Discussion

### 3.1. Nutritional Contents

Table 1 presents the proximate composition of cookies substituted with STLP at 2.5%, 5% and 10%. The moisture content of all STLP-substituted cookies decreased significantly relative to the control (*p* ≤ 0.05). Among the STLP cookies, the 5% and 10% formulations had significantly higher moisture than the 2.5% formulation (*p* ≤ 0.05). Moisture content did not change consistently with substitution level, a pattern also reported in other leaf-powder cookie studies [6,9]. Protein, dietary fiber, and ash contents increased with STLP substitution (*p* ≤ 0.05), indicating that STLP enriched the composition of the cookies compared with wheat flour alone. The increases in protein and ash were modest, whereas dietary fiber increased substantially, by roughly 5- to 6.5-fold, reflecting the fiber-rich nature of the leaf material. Similar compositional enrichment has been reported for cookies substituted with mulberry leaf powder [9] and *Moringa oleifera* leaf powder [15].

Total carbohydrate content was significantly lower only in the 10% STLP cookies relative to the control (*p* ≤ 0.05). This likely reflects the greater replacement of starch-rich wheat flour at the 10% level. No significant differences in energy emerged among cookie formulations (*p* > 0.05). The absence of significant differences in energy content indicates that STLP substitution did not meaningfully alter the caloric value of the cookies. Given that fat content in STLP was less than 0.01 g/100 g, there was no significant difference in fat among formulations (*p* > 0.05).

### 3.2. Spread Ratio, Baking Loss and Hardness

**Figure 1:**
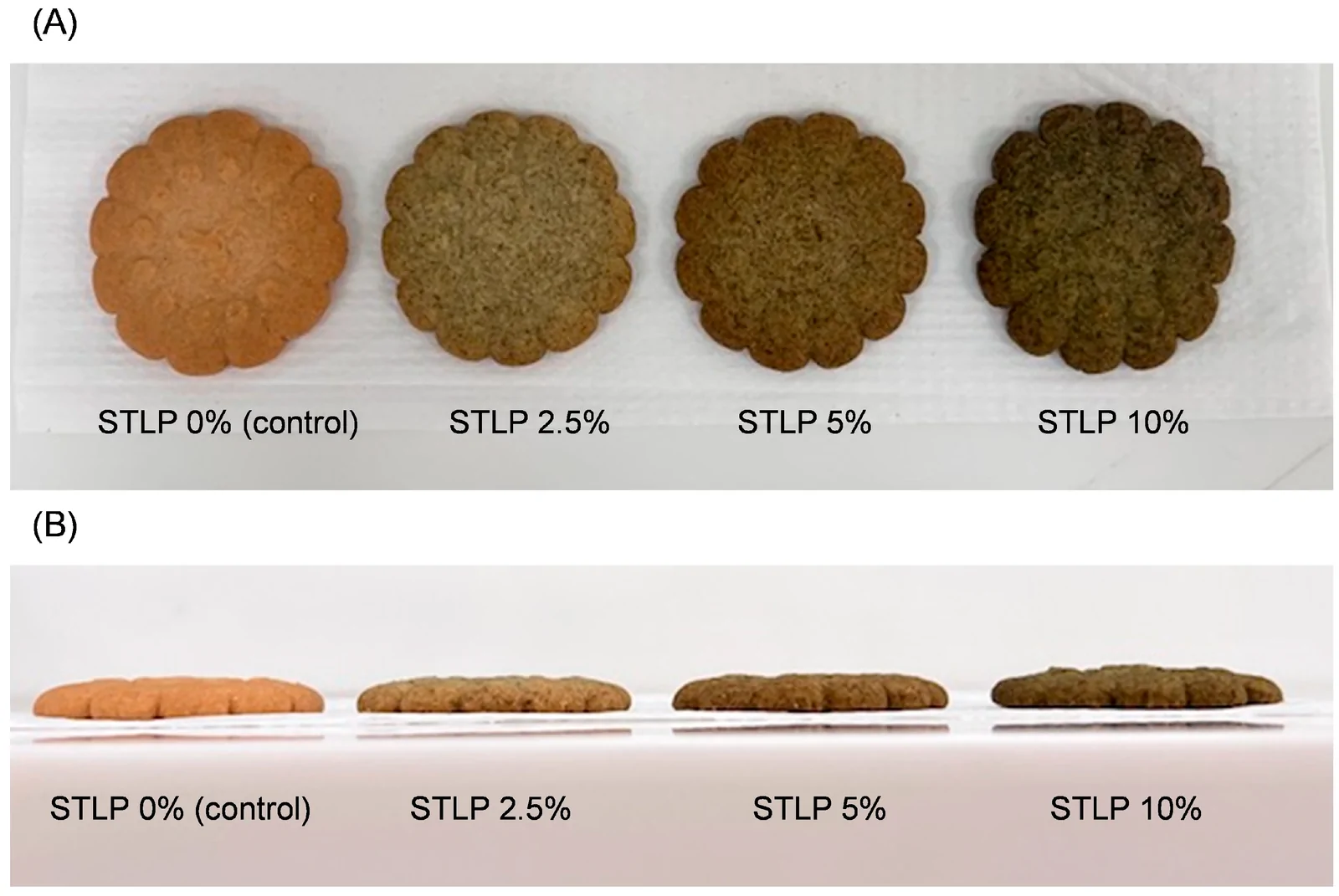
Representative photographs of cookie samples containing different levels of sacha inchi tea leaf powder (STLP): 0% STLP (control), 2.5% STLP, 5% STLP, and 10% STLP. Images show the cookies from (**A**) the top view and (**B**) the side view.

The overall appearances of cookie samples are illustrated in Figure 1A,B. The effects of partial wheat flour replacement with STLP on spread ratio, baking loss and hardness are summarized in Table 2. The spread ratio increased significantly with 10% STLP substitution, corresponding to a greater cookie diameter and lower thickness (*p* ≤ 0.05). A similar increase in spread ratio has been reported for cookies substituted with black soybean flour [8] and *Moringa oleifera* leaf powder [15]. In contrast, a previous study incorporating blueberry leaf powder into cookies found the spread ratio rose through a reduction in both diameter and thickness [10], illustrating that the dimensional basis of increased spread varies between materials. The increased spread ratio in the current study may be partly explained by the dilution of gluten-forming wheat proteins in the dough. Although the total protein content increased with STLP addition, the proportion of wheat-derived gluten-forming proteins was reduced. This reduction may have weakened the gluten network, lowering resistance to lateral expansion during baking and allowing greater spread before structural setting occurred [5,8,27]. The increased fiber content of STLP might be expected to restrict spread, as reported for other substituted cookies where spread was reduced through fiber-related dough stiffening [27] or reduced water availability during baking [22]. However, the net increase in spread observed here suggests that gluten dilution outweighed this effect within the tested range. Rheological and microstructural studies would be needed to confirm the balance of these competing influences.

Baking loss and hardness followed the same pattern as spread ratio, with significant changes observed only at 10% STLP substitution. The 10% STLP cookies showed significantly lower baking loss and higher hardness than the control (*p* ≤ 0.05), whereas the 2.5% and 5% STLP cookies did not differ markedly from the control for these parameters (*p* > 0.05). Together, these results indicate that the cookie matrix tolerated substitution up to 5% without measurable changes in physical properties, whereas 10% substitution produced more pronounced structural changes. The higher hardness observed at 10% STLP may be associated with the increased protein and dietary fiber contents, which can contribute to a firmer baked structure. Previous studies have reported that increased protein content may be associated with harder cookies, while dietary fiber can disrupt the continuity of the protein–starch matrix [8,9]. Insoluble fiber, in particular, may act as rigid filler particles that reinforce the baked matrix, as reported for cookies enriched with blueberry leaf powder [10]. The higher fiber content may also have limited water evaporation during baking through water-binding effects, thereby reducing baking loss. A comparable reduction in baking loss has been reported in cookies enriched with black soybean flour and attributed to greater water-retention affinity of such ingredients during baking [8]. Although the 5% and 10% formulations retained more moisture than the 2.5% formulation (Table 1), the 10% cookies were harder rather than softer. This apparent paradox suggests that part of the retained moisture was bound within the fiber-rich matrix rather than freely available to plasticize the structure, consistent with the anti-plasticizing effect of water in low-moisture food systems [28]. Collectively, the greater spread, increased firmness, and reduced baking loss observed at 10% STLP indicate a substitution threshold beyond which product structure is more substantially altered, which may contribute to less desirable sensory characteristics at this substitution level.

### 3.3. Color Profile

Table 2 shows the color values of the STLP-containing cookies. Lightness (L*) and yellowness (b*) declined progressively as STLP substitution increased (*p* ≤ 0.05), while all STLP cookies had significantly lower a* values as compared to (*p* ≤ 0.05), reflecting a shift toward green. As illustrated in Figure 1A,B, cookies became darker and greener at higher STLP levels. Color was measured on ground, homogenized samples rather than the intact surface, to minimize variability from position-dependent surface heterogeneity such as uneven browning between the edge and center of the cookie. Measuring color on an intact surface captures what a consumer visually perceives, whereas measuring color on a homogenized sample captures the average color of the whole cookie. Both are recognized approaches in cookie color assessment, but they are not interchangeable [29]. Therefore, the present results should be interpreted as reflecting the overall rather than the surface-specific color of the cookies.

The decrease in lightness is consistent with previous reports on flour substitution with leaf powders and non-wheat materials [6,8,9,10]. This color change may be attributed to both the intrinsic dark-green color of STLP and changes occurring during baking. The higher protein content of STLP-containing cookies may have contributed to Maillard browning, resulting in darker products [8,30]. In addition, the natural pigments present in the leaf powder likely contributed to the lower L* and b* values. Similar darkening with increasing substitution levels has been reported for cookies supplemented with *Moringa oleifera* dried leaves [6], mulberry leaf powder [9] and blueberry leaf powder [10].

All STLP cookies were significantly greener than the control. The greener appearance of the STLP cookies is likely related to chlorophyll and other green pigments in the leaf material, which can alter the characteristic color of baked products [9]. However, chlorophyll is heat-sensitive, and heating during baking can convert it to derivatives such as pheophytin, shifting the color from green toward olive-green [31]. A comparable reduction in lightness, redness, and yellowness has been reported for other leaf-powder-fortified products subjected to thermal processing [11]. Greenness did not follow a clear dose-dependent trend among the STLP levels, likely reflecting competing factors beyond protein content alone. As STLP replaces wheat flour, the pale flour background is progressively reduced, while chlorophyll from the leaf material increases greenness. This greenness may be simultaneously offset by Maillard browning, driven by the higher protein content and reducing sugars, and by the conversion of chlorophyll to pheophytin and related olive-brown degradation products during baking. Differences in moisture content among the formulations may also have influenced the perceived surface color. On the intact surface (Figure 1A), cookies may therefore appear less green at higher substitution levels despite a greater total pigment content, whereas the bulk a* values showed all STLP cookies to be greener than the control without a clear dose-dependent trend. Pigment-specific and surface-specific measurements would be needed to fully resolve these contributions.

The total color difference (Δ*E*) relative to the control increased significantly with STLP level (*p* ≤ 0.05). With all Δ*E* values above 5, the color differences would be clearly perceptible to the human eye [31]. These pronounced color changes are relevant not only to product appearance but also to sensory acceptability, as consumers may perceive darker or greener cookies as less typical of conventional bakery products.

### 3.4. Total Phenolic Content and Antioxidant Capacity

Total phenolic content and antioxidant capacity, assessed by the FRAP assay in water extracts of the cookies, are presented in Table 2. Water was selected as the extraction solvent for practicality. It also reflects the aqueous form in which STLP is traditionally consumed as a brewed tea, offering a more food-relevant estimate of extractable phenolic content than an organic solvent. The results showed that STLP incorporation progressively increased TPC relative to the control (*p* ≤ 0.05). This dose-dependent increase is consistent with the contribution of phenolic compounds from STLP. Previous studies have reported that different solvent extracts of sacha inchi leaves contain phenolic compounds and flavonoids and exhibit antioxidant activity [4,32], supporting the role of STLP as a phenolic-contributing ingredient in the cookie matrix. Similar increases in phenolic content following plant-material substitution have also been reported in other baked products [9,10,33].

FRAP values followed a similar upward pattern. Relative to the control, FRAP was significantly higher at 5% and 10% STLP substitution (*p* ≤ 0.05), whereas the 2.5% formulation showed no significant difference (*p* > 0.05). Increased FRAP values in a similar manner were previously reported in cookies substituted with *Moringa oleifera* leaf powder [15]. The FRAP assay measures the capacity of a sample to reduce ferric to ferrous ions, reflecting chemical reducing power rather than biological antioxidant activity [34]. This suggests that a minimum level of STLP incorporation may be required to produce a measurable increase in ferric-reducing capacity under the extraction and assay conditions used. Together, the increases in TPC and FRAP indicate that STLP substitution enhanced the phenolic content and reduced antioxidant capacity of cookies, particularly at the 5% and 10% levels. As hydroalcoholic solvents such as methanol are generally more efficient at extracting polyphenols, including fractions weakly bound to the food matrix, the TPC and FRAP values reported here likely represent the water-extractable rather than the total phenolic content of the cookies. These results should also be interpreted as chemical reducing capacity rather than direct evidence of biological antioxidant effects in vivo.

### 3.5. Starch Digestibility

**Figure 2:**
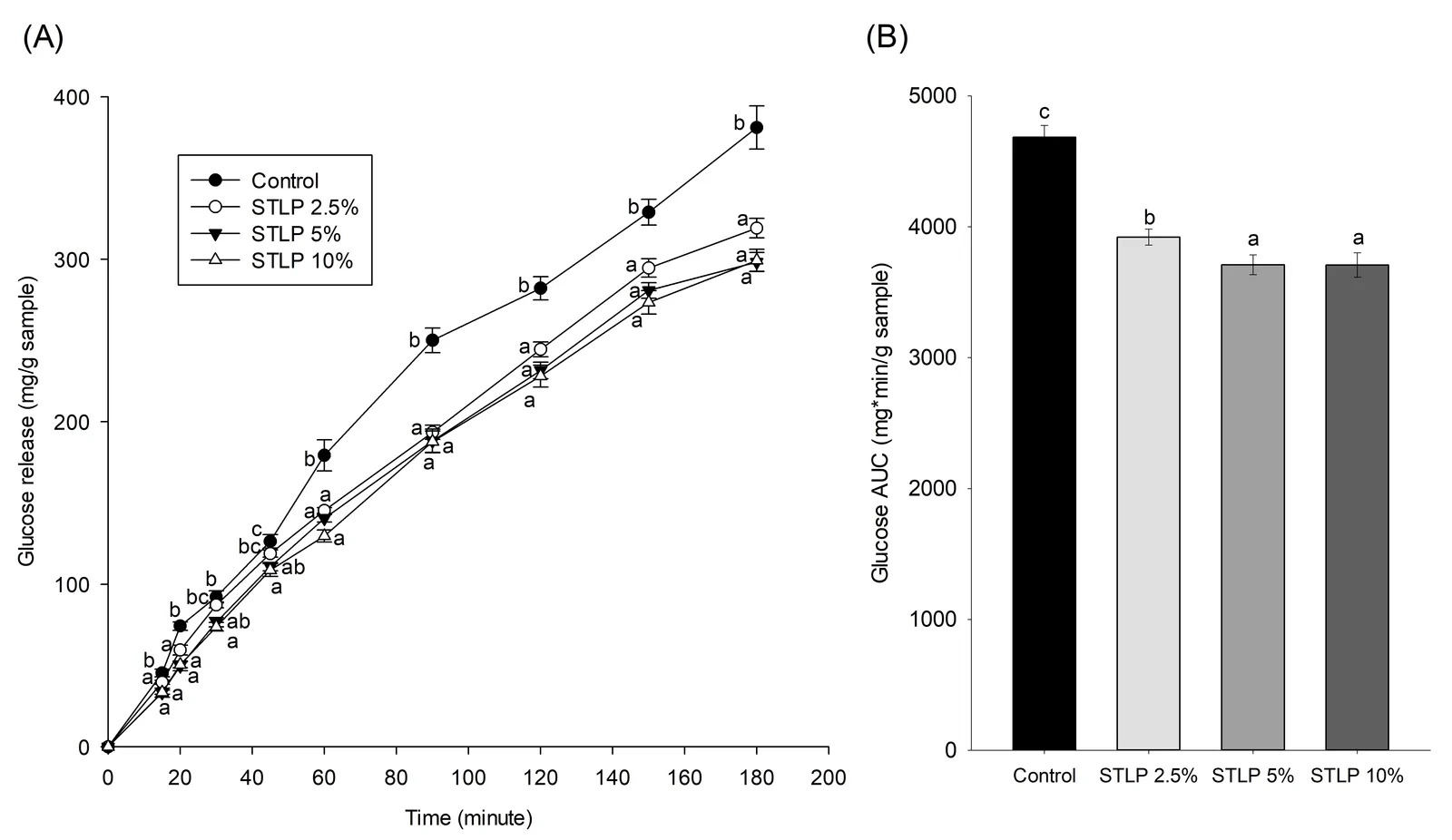
Effects of partial wheat flour substitution with sacha inchi tea leaf powder (STLP) on (**A**) glucose release during simulated in vitro digestion and (**B**) glucose area under the curve (AUC). Data represent mean ± SEM (*n* = 3). Data points with different superscript letters differ significantly among formulations at the same digestion time point in (**A**) and among AUC values in (**B**) (*p* ≤ 0.05). STLP, sacha inchi tea leaf powder; AUC, area under the curve.

Glucose release from the cookies during simulated digestion is shown in Figure 2A. Partial replacement of wheat flour with 2.5–10% STLP significantly reduced glucose release from 45 min onward relative to the control (*p* ≤ 0.05). Consistent with this finding, glucose area under the curve (AUC) decreased significantly in all STLP-substituted cookies relative to the control (Figure 2B; *p* ≤ 0.05). A similar pattern was observed for the hydrolysis index (HI) and predicted glycemic index (pGI). Both values were significantly reduced in all STLP formulations relative to the control (*p* ≤ 0.05), with no significant differences observed among the STLP substitution levels (*p* > 0.05; Table 3).

These findings suggest that STLP substitution reduced the rate and extent of starch hydrolysis under the simulated in vitro digestion conditions. Although the underlying mechanisms were not directly evaluated in the present study, several factors reported in the literature may explain this effect. Dietary fiber can reduce starch digestibility by limiting enzyme access to starch, competing for water during starch gelatinization, and modifying the structure of the food matrix [12,35]. Cellulose, a major insoluble fiber component of plant cell walls and commonly present in leaf materials, may also influence starch digestion by interacting with digestive enzymes or restricting substrate accessibility [36,37]. It has been shown to directly bind and inhibit α-amylase [12,35]. In baked matrices, dietary fiber may disrupt the continuity of the gluten–starch network and alter starch granule exposure, thereby reducing starch gelatinization and enzymatic hydrolysis [13].

By analogy with these reports, the increased dietary fiber and protein contents of the STLP cookies could have contributed to a more restrictive food matrix that limited enzyme diffusion and substrate accessibility during digestion [8,35]. Phenolic compounds contributed by STLP may additionally reduce starch hydrolysis through inhibition of digestive enzymes. Tea made from *Camellia sinensis* and sacha inchi leaf has been shown to inhibit α-glucosidase [38]. Nevertheless, enzyme inhibition, starch gelatinization, and microstructural changes were not directly measured in the present study. Therefore, the proposed mechanisms should be regarded as plausible explanations rather than confirmed pathways. Moreover, the pGI values were derived from an in vitro starch digestion model and should not be interpreted as direct evidence of postprandial glycemic responses in humans.

### 3.6. Sensory Acceptability

The sensory rating of the cookie samples is summarized in Table 4. The participants were mostly female (82%), with a mean age of 25.72 ± 6.73 years. Compared with the control, STLP-substituted cookies had significantly lower scores for appearance, color, smell, flavor, and overall acceptability (*p* ≤ 0.05), whereas texture scores did not differ significantly among formulations. The 10% STLP cookies generally received the lowest sensory ratings (*p* ≤ 0.05).

Appearance and color scores decreased as the STLP substitution level increased. The instrumental color measurements showed that STLP-substituted cookies were darker and greener than the yellowish-brown control, with differences large enough to be clearly perceptible to the human eye based on Δ*E* values greater than 5 (Figure 1, Table 2). The darker color may be related to Maillard browning, promoted partly by the higher protein content of STLP cookies, together with the intrinsic color of the leaf powder. The greener appearance is consistent with pigments from the leaf material, particularly chlorophyll-related compounds [9]. The progressive shift away from the typical color of conventional cookies may have negatively affected visual acceptance, especially at the higher substitution level. Although color was measured on homogenized samples rather than the intact surface, these instrumental measurements should be interpreted as reflecting the overall color of the cookie rather than its surface-specific appearance. Similar reductions in consumer acceptance have been reported for cookies enriched with *Moringa oleifera* green leaf powders, where increasing greenness and deviation from expected product appearance reduced preference [6].

Smell, flavor, and overall acceptability also decreased with STLP incorporation. The lower aroma scores may be associated with the grassy or herbal notes characteristic of leaf-derived ingredients, which may have been carried over into the final product. Because aroma strongly contributes to flavor perception, these sensory characteristics may also explain the lower flavor and overall acceptability scores of STLP-containing cookies. For overall acceptability, the 5% formulation scored significantly higher than the 10% formulation, indicating that moderate substitution may provide a more favorable balance between compositional improvement and sensory quality.

In contrast, texture did not differ significantly among formulations, although the 10% STLP cookies had the numerically lowest texture score. In the instrumental analysis, hardness increased significantly only at the 10% level. Based on principal component analysis, a recent study showed that higher substitution levels of blueberry leaf powder (5–7.5%) were strongly associated with hardness and herbaceous smell, while lower substitution levels (2.5%) were positively associated with overall acceptability [10]. This parallels the present study, in which overall acceptability was significantly higher at 5% than at 10% STLP, consistent with reduced acceptability as substitution increased.

Overall, these findings indicate that STLP substitution affected visual and flavor-related attributes more strongly than perceived texture within the tested range. Further studies should explore techniques such as encapsulation or flavor-masking with compatible ingredients to reduce grassy notes and stabilize color, while aiming to preserve the functional compounds of the cookies.

### 3.7. Limitations and Future Perspectives

Several limitations should be acknowledged. First, the proposed role of bound water and water distribution in textural changes was inferred from composition and baking behavior rather than directly measured. Future studies using water activity analysis, low-field nuclear magnetic resonance, or differential scanning calorimetry could clarify water mobility and thermal transitions in STLP-containing cookie matrices. In addition, texture was assessed using hardness as the primary parameter. Measuring further parameters such as fracturability and crispness would provide a more complete description of the textural changes associated with STLP substitution. Second, only total dietary fiber was quantified. Analysis of soluble and insoluble fiber fractions would improve interpretation of cookie structure and starch digestibility. Third, the mechanisms underlying reduced starch hydrolysis were not directly examined. Assessments of starch gelatinization, microstructure, and digestive enzyme inhibition would strengthen mechanistic understanding. Fourth, starch digestibility and predicted glycemic index were assessed using an in vitro model; therefore, human postprandial studies are needed to confirm translational relevance. Fifth, the characterization of STLP was limited. Although the proximate composition of STLP was determined using a representative sample, these measurements were not performed with sufficient independent replication. In addition, the phenolic content and particle-size distribution of STLP were not characterized. These limitations may restrict the interpretation of how the intrinsic properties of STLP contributed to the physicochemical and digestibility-related characteristics of the cookies. Future studies should therefore include comprehensive characterization of STLP using adequately replicated analyses to improve the interpretation and reproducibility of the findings. Finally, although STLP improved selected compositional and in vitro digestibility-related properties, it reduced sensory acceptability. The sensory evaluation was based on affective testing using a 9-point hedonic scale. Descriptive sensory analysis using a trained panel would provide more objective profiling of attributes such as color intensity and grassy or herbal notes. In addition, the panel was unbalanced in sex composition (9 males and 41 females), which may have influenced the acceptability ratings, and panelists were not screened for cookie consumption frequency. A larger and more balanced panel including regular consumers of the product would improve the generalizability of the sensory findings. Further optimization of STLP level, particle size, color, flavor, and aroma may improve consumer acceptance and practical application in bakery products.

## 4. Conclusions

Substituting part of the wheat flour with sacha inchi tea leaf powder (STLP) improved selected compositional and antioxidant-related properties of cookies, as shown by increased protein, dietary fiber, ash, total phenolic content, and ferric-reducing antioxidant power. Glucose release, the hydrolysis index, and the predicted glycemic index were lower with STLP substitution under simulated in vitro digestion, suggesting slower starch hydrolysis in the tested model. However, higher STLP substitution, particularly at 10%, altered the physical properties of the cookies and reduced several sensory attributes.

Among the substitution levels tested, 5% STLP appeared to offer a reasonable balance between functional enhancement and sensory quality. At this level, the cookies showed significant increases in dietary fiber, protein, phenolic content, and ferric-reducing capacity relative to the control, together with a reduction in in vitro starch digestibility comparable to that at 10% substitution, while retaining significantly higher appearance, color, and overall acceptability scores than the 10% formulation. Lower substitution (2.5%) did not significantly enhance antioxidant capacity, whereas 10% produced the greatest functional improvements but lower sensory acceptability.

Overall, these findings indicate that STLP has potential as a plant-derived ingredient for cookie reformulation, with 5% substitution representing a practical balance between nutritional enhancement and sensory quality. For industrial application, the use of STLP could support the valorization of an underutilized agricultural material and the development of fiber- and antioxidant-enriched cookies, although further optimization of formulation and processing would be needed to improve sensory acceptability and ensure consistent product quality at scale.

## Figures and Tables

**Table 1:** Proximate analysis and energy content of cookies partially substituted with sacha inchi tea leaf powder.

Parameter (per 100 g)	Control	STLP 2.5%	STLP 5%	STLP 10%
Energy (kcal)	520.12 ± 0.96 ^a^	523.19 ± 0.72 ^a^	521.83 ± 0.23 ^a^	520.76 ± 0.58 ^a^
Moisture (g)	2.44 ± 0.03 ^c^	1.77 ± 0.03 ^a^	2.05 ± 0.01 ^b^	2.13 ± 0.05 ^b^
Protein (g)	6.17 ± 0.03 ^a^	6.38 ± 0.02 ^b^	6.54 ± 0.05 ^b^	6.74 ± 0.03 ^c^
Total fat (g)	26.70 ± 0.22 ^a^	26.94 ± 0.16 ^a^	27.03 ± 0.07 ^a^	27.14 ± 0.16 ^a^
Total carbohydrate (g)	63.80 ± 0.27 ^b^	63.94 ± 0.20 ^b^	63.11 ± 0.04 ^ab^	62.38 ± 0.25 ^a^
Dietary fiber (g)	0.27 ± 0.01 ^a^	1.65 ± 0.04 ^b^	2.09 ± 0.02 ^c^	2.08 ± 0.05 ^c^
Ash (g)	0.90 ± 0.00 ^a^	1.12 ± 0.01 ^b^	1.28 ± 0.02 ^c^	1.60 ± 0.01 ^d^

Data represent mean ± SEM (*n* = 2). Within each row, values with different superscript letters differ significantly among formulations (*p* ≤ 0.05). STLP, sacha inchi tea leaf powder.

**Table 2:** Physical, color, phenolic, and antioxidant properties of cookies partially substituted with sacha inchi tea leaf powder.

Parameter	Control	STLP 2.5%	STLP 5%	STLP 10%
Diameter (cm)	4.69 ± 0.01 ^a^	4.72 ± 0.02 ^a^	4.74 ± 0.01 ^a^	4.94 ± 0.03 ^b^
Thickness (cm)	0.70 ± 0.00 ^b^	0.68 ± 0.00 ^b^	0.68 ± 0.00 ^b^	0.66 ± 0.01 ^a^
Spread ratio	6.72 ± 0.03 ^a^	6.94 ± 0.05 ^a^	6.94 ± 0.04 ^a^	7.49 ± 0.10 ^b^
Baking loss (%)	3.40 ± 0.04 ^b^	3.30 ± 0.09 ^b^	3.24 ± 0.09 ^b^	2.75 ± 0.07 ^a^
Hardness (g)	752.26 ± 18.72 ^a^	751.63 ± 15.53 ^a^	730.69 ± 18.22 ^a^	1358.95 ± 19.33 ^b^
L*	26.61 ± 0.36 ^d^	22.59 ± 0.56 ^c^	16.22 ± 0.65 ^b^	12.60 ± 0.30 ^a^
a*	9.70 ± 0.08 ^c^	2.71 ± 0.15 ^a^	3.67 ± 0.03 ^b^	2.42 ± 0.04 ^a^
b*	21.68 ± 0.11 ^d^	18.69 ± 0.40 ^c^	16.50 ± 0.31 ^b^	13.25 ± 0.12 ^a^
**∆** *E*	-	8.67 ± 0.37 ^a^	13.11 ± 0.86 ^b^	17.92 ± 0.91 ^c^
TPC (mg GAE/g sample)	0.36 ± 0.02 ^a^	0.43 ± 0.02 ^a^	0.53 ± 0.03 ^b^	0.74 ± 0.02 ^c^
FRAP (mM FeSO_4_ equivalent/g sample)	0.28 ± 0.05 ^ab^	0.22 ± 0.01 ^a^	0.36 ± 0.02 ^b^	0.50 ± 0.02 ^c^

Data represent mean ± SEM (*n* = 3), which were measured using 20 cookies per formulation. Within each row, values with different superscript letters differ significantly among formulations (*p* ≤ 0.05). Δ*E* was calculated relative to the control cookies. STLP, sacha inchi tea leaf powder; TPC, total phenolic content; GAE, gallic acid equivalent; FRAP, ferric-reducing antioxidant power.

**Table 3:** Hydrolysis index and predicted glycemic index of cookies partially substituted with sacha inchi tea leaf powder based on in vitro starch digestion.

Parameter	Control	STLP 2.5%	STLP 5%	STLP 10%
Hydrolysis index (%)	32.36 ± 0.63 ^b^	27.09 ± 0.42 ^a^	25.63 ± 0.52 ^a^	25.61 ± 0.64 ^a^
Predicted glycemic index	57.47 ± 0.60 ^b^	54.58 ± 0.40 ^a^	53.78 ± 0.50 ^a^	53.77 ± 0.61 ^a^

Data represent mean ± SEM (*n* = 3). Within each row, values with different superscript letters differ significantly among formulations (*p* ≤ 0.05). HI, hydrolysis index; pGI, predicted glycemic index; STLP, sacha inchi tea leaf powder. The pGI values were calculated from in vitro starch hydrolysis data and do not represent in vivo glycemic index values.

**Table 4:** Sensory acceptability of cookies partially substituted with sacha inchi tea leaf powder.

Parameter	Control	STLP 2.5%	STLP 5%	STLP 10%
Appearance	7.64 ± 0.19 ^c^	6.94 ± 0.25 ^b^	6.70 ± 0.22 ^b^	5.44 ± 0.27 ^a^
Color	7.50 ± 0.22 ^c^	6.54 ± 0.27 ^b^	6.20 ± 0.22 ^b^	5.04 ± 0.29 ^a^
Texture	7.10 ± 0.22 ^a^	6.90 ± 0.18 ^a^	7.06 ± 0.19 ^a^	6.50 ± 0.26 ^a^
Smell	6.72 ± 0.18 ^b^	5.20 ± 0.25 ^a^	5.68 ± 0.25 ^a^	4.96 ± 0.27 ^a^
Flavor	7.28 ± 0.17 ^b^	5.66 ± 0.29 ^a^	6.06 ± 0.28 ^a^	5.34 ± 0.29 ^a^
Overall acceptability	7.42 ± 0.15 ^c^	5.88 ± 0.24 ^ab^	6.34 ± 0.24 ^b^	5.32 ± 0.24 ^a^

Data represent mean ± SEM (*n* = 50 participants). Within each row, values with different superscript letters differ significantly among formulations (*p* ≤ 0.05). Sensory attributes were rated on a 9-point hedonic scale (1 = extremely dislike, 5 = neither like nor dislike, 9 = extremely like). STLP, sacha inchi tea leaf powder.

## Data Availability

The original contributions presented in this study are included in the article. Further inquiries can be directed to the corresponding author.

## Abbreviations

The following abbreviations are used in this manuscript:

STLP	sacha inchi tea leaf powder
TPTZ	2,4,6-tripyridyl-s-triazine
AOAC	Association of Official Analytical Chemists
TPC	total phenolic content
FRAP	ferric-reducing antioxidant power
GAE	gallic acid equivalent
AUC	area under the curve
HI	hydrolysis index
pGI	predicted glycemic index
SEM	standard error of the mean
